# The Histidine Kinase BinK Is a Negative Regulator of Biofilm Formation and Squid Colonization

**DOI:** 10.1128/JB.00037-16

**Published:** 2016-09-09

**Authors:** John F. Brooks, Mark J. Mandel

**Affiliations:** Department of Microbiology-Immunology, Northwestern University Feinberg School of Medicine, Chicago, Illinois, USA; Geisel School of Medicine at Dartmouth

## Abstract

Bacterial colonization of animal epithelial tissue is a dynamic process that relies on precise molecular communication. Colonization of Euprymna scolopes bobtail squid by Vibrio fischeri bacteria requires bacterial aggregation in host mucus as the symbiont transitions from a planktonic lifestyle in seawater to a biofilm-associated state in the host. We have identified a gene, *binK* (biofilm inhibitor kinase; VF_A0360), which encodes an orphan hybrid histidine kinase that negatively regulates the V. fischeri symbiotic biofilm (Syp) *in vivo* and *in vitro*. We identified *binK* mutants as exhibiting a colonization advantage in a global genetic screen, a phenotype that we confirmed in controlled competition experiments. Bacterial biofilm aggregates in the host are larger in strains lacking BinK, whereas overexpression of BinK suppresses biofilm formation and squid colonization. Signaling through BinK is required for temperature modulation of biofilm formation at 28°C. Furthermore, we present evidence that BinK acts upstream of SypG, the σ^54^-dependent transcriptional regulator of the syp biofilm locus. The BinK effects are dependent on intact signaling in the RscS-Syp biofilm pathway. Therefore, we propose that BinK antagonizes the signal from RscS and serves as an integral component in V. fischeri biofilm regulation.

**IMPORTANCE** Bacterial lifestyle transitions underlie the colonization of animal hosts from environmental reservoirs. Formation of matrix-enclosed, surface-associated aggregates (biofilms) is common in beneficial and pathogenic associations, but investigating the genetic basis of biofilm development in live animal hosts remains a significant challenge. Using the bobtail squid light organ as a model, we analyzed putative colonization factors and identified a histidine kinase that negatively regulates biofilm formation at the host interface. This work reveals a novel *in vivo* biofilm regulator that influences the transition of bacteria from their planktonic state in seawater to tight aggregates of cells in the host. The study enriches our understanding of biofilm regulation and beneficial colonization by an animal's microbiome.

## INTRODUCTION

During colonization of animal tissue, communication between colonizing bacteria and the host regulates processes in both organisms that influence the outcome of the interaction. The host innate immune system senses bacterial products and plays a major role in delimiting the species and strains of bacteria that can colonize specific niches. In turn, bacteria release products that modulate host responses and produce signaling proteins that respond at the host interface (1). To understand the impact of the molecular dialogue that occurs between colonizing bacteria and their animal hosts, it is useful to examine a reduced system in which individual steps can be isolated. Colonization of the Hawaiian bobtail squid Euprymna scolopes by the luminous Gram-negative bacterium Vibrio fischeri provides such a system (2, 3). V. fischeri is the only species that colonizes the squid light organ despite the large abundance of bacteria in seawater (approximately 10^6^ bacteria per milliliter) and the proportionally low representation of V. fischeri in the seawater population (0.02% as a high estimate) (4). A powerful combination of bacterial genetics and direct imaging at the host-microbe interface has allowed for the mapping of specific stages of symbiotic colonization with high spatial and temporal resolution (5, 6). During initiation of the symbiosis, bacterial peptidoglycan stimulates host mucus secretion, which harvests bacteria from the seawater (7). The bacteria adhere in the mucus, and V. fischeri bind host cilia and aggregate in a process that requires biofilm development through production of the symbiosis polysaccharide (Syp) (8). Biofilm development is required for robust colonization of the animal and for host colonization specificity (9, 10). After the biofilm aggregate is formed, the bacteria migrate through the host mucus and predominate over other bacteria through a process that does not require flagellar motility or chemotaxis (11–13). The bacteria proceed to use flagellar motility and chemotaxis to migrate through one of the six pores into an internal crypt of the light organ, completing initiation of the symbiosis (5, 12, 14). Bacterial products, including the peptidoglycan fragment tracheal cytotoxin and the lipopolysaccharide (LPS), are shed by the colonizing V. fischeri and lead to apoptosis and regression of the host tissue that recruits the symbiont (7, 15, 16). Overall, this highly selective process of colonization initiation requires proper temporal and spatial regulation of bacterial behaviors necessary for efficient colonization of the squid host.

Many bacterial behaviors required for robust colonization of the squid host—biofilm formation, flagellar motility, and chemotaxis—are governed by two-component signaling (TCS) (17). TCS systems are prevalent throughout bacteria and enable the coupling of environmental stimulus perception with appropriate behavioral outputs (18–20). TCS systems include a sensor histidine kinase (HK) and a response regulator (RR) that effects an output, and often variations of TCS are connected in more complicated circuitry termed a phosphorelay (21–23). Usually cognate HK-RR pairs are encoded adjacent to each other in the genome; in other cases an “orphan” HK or RR does not have a known partner.

TCS systems play important roles during squid colonization by V. fischeri (17). Light organ pore entry requires biogenesis of its polar flagella and chemotaxis, which rely on the HK-RR pairs CheA-CheY, CheA-CheB, and FlrB-FlrC (5, 12). Biofilm development involves the HKs RscS and SypF. RscS, a hybrid histidine kinase, is proposed to autophosphorylate within its dimerization and histidine phosphotransferase (DHp) domain and transfer the phosphate to the conserved aspartate residue within the receiver (REC) domain. Although RscS contains a histidine phosphotransferase (HPt) domain, there is convincing evidence that the phosphorylation signal from RscS instead uses the HPt domain of SypF (24). SypF then phosphorylates two RRs, SypG and SypE, which act to positively and negatively regulate biofilm formation, respectively (24–26).

In a previous report we conducted a high-throughput, global analysis of V. fischeri factors required for squid colonization (6). Given the importance of HKs in regulating bacterial behaviors in the host environment, we examined the HKs in the global data set. HKs known to be important for robust colonization of the animal exhibited significant competitive defects, validating this analysis. An unstudied HK, VF_A0360, exhibited a dramatic competitive advantage upon gene interruption. We report here that VF_A0360 (i) negatively regulates aggregation behavior in the squid host environment, (ii) inhibits Syp polysaccharide production and biofilm phenotypes, and (iii) reduces syp transcriptional regulation. Together these data identify VF_A0360 as a negative regulator of Syp polysaccharide production. Following the nomenclature in Bassis and Visick (27) we have named VF_A0360 as *binK*, for biofilm inhibitor-kinase, and we use the *binK* nomenclature for the remainder of this report.

## MATERIALS AND METHODS

### Bacterial strains, plasmids, and media.

V. fischeri and Escherichia coli strains used in this study are listed in Table 1. V. fischeri strains were grown at 25°C in Luria-Bertani salt (LBS) medium (per liter, 10 g Bacto Tryptone, 5 g yeast extract and 20 g NaCl, 50 ml 1 M Tris buffer [pH 7.5], in distilled water). E. coli strains, as used for cloning and conjugation, were grown at 37°C in Luria-Bertani (LB) medium (28). When necessary, antibiotics were added to the media at the following concentrations: tetracycline, 5 μg/ml for V. fischeri; erythromycin, 5 μg/ml for V. fischeri; kanamycin, 100 μg/ml for V. fischeri and 50 μg/ml for E. coli; and chloramphenicol, 5 μg/ml for V. fischeri and 25 μg/ml for E. coli. Growth media were solidified with 1.5% agar as needed. Standard microbial techniques were used to mobilize plasmids into V. fischeri strains (28). In brief, the *binK* alleles used in this report were generated by PCR amplification and cloned into either the mobilizable vector pVSV104 or the mini-Tn*7* delivery vector pEVS107. Constructs generated using the pVSV104 vector backbone were introduced into V. fischeri by triparental mating (100-μl aliquots of each pEVS104-containing helper, pVSV104-type-containing donor, and V. fischeri recipient were mixed), and constructs generated using the pEVS107 vector backbone were introduced into V. fischeri by tetraparental mating (100-μl aliquots of each pEVS104-containing helper, pUX-BF13-containing transposase, pEVS107-type plasmid-containing donor, and V. fischeri recipient were mixed) (29).

**TABLE 1:** Bacterial strains and plasmids

Strain or plasmid	Relevant genotype or description^*a*^	Reference(s)^*b*^
Vibrio fischeri strains		
MJM1100	ES114	54, 55
MJM1107	MJM1100/pVSV102	6
MJM1198	MJM1100 rscS*	37
MJM1575	MJM1100/pVSV103	
MJM1778	MJM1100 rscS*/pVSV104	
MJM1782	MJM1100/pVSV104	
MJM1946	MJM1100 rscS* sypB::Tn*erm*	
MJM1952	MJM1100 rscS* sypQ::Tn*erm*	
MJM1954	MJM1100 rscS* sypG::Tn*erm*	
MJM2251	MJM1100 Δ*binK*	
MJM2255	MJM1100 rscS* Δ*binK*	
MJM2256	MJM1100 Δ*binK*/pVSV104	
MJM2265	MJM1100 Δ*binK*/pVSV102	
MJM2385	MJM1100 rscS*/pBinK	
MJM2386	MJM1100/pBinK	
MJM2387	MJM1100 Δ*binK*/pBinK	
MJM2388	MJM1100 rscS* Δ*binK*/pBinK	
MJM2389	MJM1100 rscS* Δ*binK*/pVSV104	
MJM2476	MJM1100 rscS* Δ*binK att*Tn*7*::*binK^+^*	
MJM2478	MJM1100 Δ*binK att*Tn*7*::*binK^+^*	
MJM2479	MJM1100 rscS* *att*Tn*7*::*erm*	
MJM2480	MJM1100 rscS* Δ*binK att*Tn*7*::*erm*	
MJM2487	MJM1100 rscS* Δ*binK*/pLostfoX-Kan	
MJM2488	MJM1100 rscS* Δ*binK sypG*::Tn*erm*	
MJM2489	MJM1100 rscS* *att*Tn*7*::*erm*/pM1422	
MJM2490	MJM1100 rscS* Δ*binK att*Tn*7*::*erm*/pM1422	
MJM2491	MJM1100 rscS* Δ*binK att*Tn*7*::*binK^+^*/pM1422	
MJM2492	MJM1100 rscS* *sypG*::Tn*erm*/pM1422	
MJM2495	MJM1100 Δ*binK att*Tn*7*::*binK^+^*/pVSV102	
KV3299	ES114 Δ*sypE*	46
MJM2546	KV3299/pEAH73, pVSV104	
MJM2547	KV3299/pEAH73, pBinK	
KV5479	ES114 Δ*sypA att*Tn*7*:: *sypA*^+^	45
MJM2569	KV5479/pEAH73, pVSV104	
MJM2570	KV5479/pEAH73, pBinK	
KV5481	ES114 Δ*sypA att*Tn*7*:: *sypA*^S56A^	45
MJM2573	KV5481/pEAH73, pVSV104	
MJM2574	KV5481/pEAH73, pBinK	
MJM2647	MJM1100/pKV69	
MJM2648	MJM1100/pEAH73	
MJM2649	MJM2251/pKV69	
MJM2650	MJM2251/pEAH73	
MJM2656	KV3299/pKG11, pVSV104	
MJM2658	KV3299/pKG11, pBinK	
MJM2660	KV5479/pKG11, pVSV104	
MJM2662	KV5479/pKG11, pBinK	
MJM2664	KV5481/pKG11, pVSV104	
MJM2666	KV5481/pKG11, pBinK	
Plasmids		
pEVS122	V. fischeri suicide vector (Erm^r^)	56
pUC19	Cloning vector (Amp^r^)	Lab stock
pVSV102	Constitutive GFP (Kan^r^)	34
pVSV103	Constitutive LacZ (Kan^r^)	34
pVSV104	Complementation vector (Kan^r^)	34
pEVS104	Conjugal helper plasmid (Kan^r^)	28
pEVS107	Mini-Tn*7* mobilizable vector (Erm^r^ Kan^r^)	29
pUX-BF13	Tn*7* tranposition helper (Amp^r^)	57
pKV69	Complementation vector (Cam^r^ Tet^r^)	41
pEAH73	pKV69 carrying wild-type *sypG* (Cam^r^ Tet^r^)	46
pKG11	pKV69 carrying rscS* (Cam^r^ Tet^r^)	25
pM1422	pTM267 *sypA*′-*gfp^+^* (Cam^r^)	
pBinK	pVSV104 carrying wild-type *binK* (Kan^r^)	
pTn7BinK	pEVS107 carrying wild-type *binK* (Erm^r^)	
pLostfoX-Kan	Arabinose-inducible TfoX for transformation (Kan^r^)	6

a Erm, erythromycin; Amp, ampicillin; Kan, kanamycin; Cam, chloramphenicol; Tet, tetracycline.

b Strains and plasmids with no reference listed were constructed for this study.

### DNA synthesis and sequencing.

Each of the primers listed in Table 2 was synthesized by Integrated DNA Technologies (Coralville, IA). Full inserts from all cloned constructs were verified by Sanger DNA sequencing at Northwestern University Feinberg School of Medicine Center for Genetic Medicine.

**TABLE 2:** Primer list

Name	Sequence^*a*^ (5′ to 3′)	Construct(s) (purpose[s])^*b*^
JFB_275	GAGCCTTTTAAATCCCCTAACATT	Δ*binK* (P)
JFB_276	GCCATCGATTAATGACATATTATTATTCAT	Δ*binK* (P)
JFB_277	GCCATCGATGCGTATACATAAATAATGATTC	Δ*binK* (P)
JFB_278	TTTCAATACTGTGTTTTTATGCTGT	Δ*binK* (P)
MJM-675	GCTTTCGAGCCTTTTAAA	Δ*binK* (S)
MJM-676	GTTTTTGTATTCAACACG	Δ*binK* (S)
MJM-677	CCAACAGCAAGACTTACT	Δ*binK* (S)
MJM-678	AGAGTTTATTGAATTCGG	Δ*binK* (S)
MJM-679	CAAAACGCTTATCCAAAA	Δ*binK* (S)
MJM-680	GAGGGTAAGATCAAACTT	Δ*binK* (S)
MJM-681	AGGGTGTAGATATTTGGC	Δ*binK* (S)
MJM-682	GTTGATGTAGGCTAAATG	Δ*binK* (S)
MJM-683	ACCATCAACGGCTTTGAT	Δ*binK* (S)
MJM-684	CGTTTTCAATCTTAATGC	Δ*binK* (S)
MJM-685	GCGTGGTGAGACTTCAGA	Δ*binK* (S)
JFB_287	ATGGAGTTTCTACGTCAACCAGAA	Δ*binK* (V)
JFB_288	TGTTATAACGATTACATGGCAGCG	Δ*binK* (V)
MJM-713	CTAATGACAGATGTGTATGTCAG	pBinK (P, V)
MJM-714	TTATAACGATTACATGGCAGCG	pBinK (P, V)
M13 Rev	AGCGGATAACAATTTCACACAGG	Multiple constructs (V, S)
M13 For	CGCCAGGGTTTTCCCAGTCACGAC	Multiple constructs (V, S)
JFB_355	CTATGCGGCATCAGAGCA	pTn7BinK, pBinK (S)
JFB_356	CGACGTTTTATAACGATT	pTn7BinK, pBinK (S)
JFB_357	ATTTATGTATACGCTTCC	pTn7BinK, pBinK (S)
JFB_358	CGCAAAATCCGGCCTTTT	pTn7BinK, pBinK (S)
JFB_359	AAATGATAATCGCTGGTC	pTn7BinK, pBinK (S)
JFB_360	ACCCTTTTTCTGAATCAA	pTn7BinK, pBinK (S)
JFB_361	GATGTTCATCAAGCATTA	pTn7BinK, pBinK (S)
JFB_362	GAGGTGTTCGAATTTCGT	pTn7BinK, pBinK (S)
JFB_363	GAGCGAAAGTCTCATCAG	pTn7BinK, pBinK (S)
JFB_364	AAACCTCAGACCATGAAA	pTn7BinK, pBinK (S)
JFB_365	GGAAAGAGAATGATTAAG	pTn7BinK, pBinK (S)
JFB_366	ATTCAAAGAATATGGTGC	pTn7BinK, pBinK (S)
JFB_367	ATGACCATGATTACGCCA	pTn7BinK, pBinK (S)
JFB_368	TACGACAAAGTACTTAAG	pTn7BinK, pBinK (S)
JFB_369	GTTACTCTATCGATGTTC	pTn7BinK, pBinK (S)
JFB_370	TCACCGCTTCACAACCTT	pTn7BinK, pBinK (S)
JFB_371	CTATTTTATTGGCTTGTG	pTn7BinK, pBinK (S)
JFB_372	AACTGAAACCGATTTAAC	pTn7BinK, pBinK (S)
JFB_373	ATGCCGTTAAATTTACTC	pTn7BinK, pBinK (S)
JFB_374	TTGAGGTGATTGAGCCAA	pTn7BinK, pBinK (S)
JFB_375	TTGAACGTACAATTGAAG	pTn7BinK, pBinK (S)
JFB_376	TAGATATGGTGATGAGTA	pTn7BinK, pBinK (S)
JFB_377	ACTGAATTACGTTTAACG	pTn7BinK, pBinK (S)
JFB_378	AGTGAGTCGTATTACAAT	pTn7BinK, pBinK (S)
JFB_424	GGCGCGCCTAGGGCCCTC	pTn7BinK (P)
JFB_425	TCGAGGTACCTGGCCACTAGTAGATCTCTG	pTn7BinK (P)
JFB_426	GGCCCTAGGCGCGCCGGTACCTTATAACGATTACATGGCAGC	pTn7BinK (P)
JFB_427	GGCCAGGTACCTCGAGGTACCCTAATGACAGATGTGTATGTC	pTn7BinK (P)
Tn7 Site F	TGTTGATGATACCATTGAAGCTAAA	*att*Tn*7*::*binK*^+^ (V)
Tn7 Site R	CTTGCTGTATGTATTTGCTGATGA	*att*Tn*7*::*binK*^+^ (V)
pEVS107 F	ACCTATCAAGGTGTACTGCCTTCC	pTn7BinK (V)
pEVS107 R	GTCGTTAAATGCCCTTTACCTGT	pTn7BinK (V)
JFB_379	GATAGCATTTTGAATGACTTCACG	*sypG*::Tn*erm* (V)
MJM-477	TTCCATAACTTCTTTTACGTTTCC	Transposon-specific primer (V)
MJM-475F	GCGCATGCCCGGGGCCTTACTTGGACACGAATCA	*sypA*′-*gfp^+^* (P, S)
MJM-476R	GCACTAGTCTAGATTAGTCCATATCACCTTGAACTGATAGC	*sypA*′-*gfp^+^* (P, S)

a ClaI restriction site is underlined.

b Primers were used for PCR during construction (P), PCR to verify insertion/orientation (V), and/or sequencing (S) to confirm the construct.

### Construction of Δ*binK* strain.

A strain lacking the *binK* gene was constructed using allelic exchange, resulting in an in-frame deletion (30, 31). Briefly, the Δ*binK* allele was generated by PCR amplifying two fragments, each approximately 1.6 kb of DNA, flanking the *binK* coding sequence. The gene fragments were amplified using 1 U Pfx50 polymerase (Invitrogen) and the reaction buffer provided, 300 μM (each) dNTP, 0.3 μM (each) primer, and 250 ng MJM1100 genomic DNA. Primers for the upstream fragment include JFB_275 and JFB_276, and primers for the downstream fragment include JFB_277 and JFB_278. The upstream and downstream fragments were blunt cloned into the vectors pUC19 and pEVS122, respectively, after digestion with SmaI. The resulting plasmid constructs were fused, creating a “megaplasmid,” using the engineered ClaI restriction enzyme sites (designated in the primers JFB_276 and JFB_277). Proper orientation of the fragments was screened with PCR using the primer pair JFB_275 and JFB_278. The megaplasmid was sequenced using the primers MJM-675 through MJM-685. Once confirmed, the megaplasmid was conjugated into V. fischeri ES114, and transconjugants were screened for double recombinants using the primer pair JFB_287 and JFB_288.

### Construction of pTn7BinK.

A gene fragment containing the *binK* allele was integrated into the *att*Tn*7* site using the mini-Tn*7* delivery vector pEVS107. PCR amplification generated a 3,196-bp fragment that included the *binK* open reading frame (2,595 kb) and 300 bp upstream and 301 bp downstream of the open reading frame. PCR was conducted using Pfx50 as described above with primers JFB_426 and JFB_427 and MJM1100 genomic DNA. The vector backbone (pEVS107) was amplified using the primer pair JFB_424 and JFB_425. The gene fragment was introduced into pEVS107 with Gibson Assembly using the Gibson Assembly master mix (New England BioLabs). The plasmid construct was sequenced using the primers JFB_355 through JFB_378 and pEVS107 F and pEVS107 R. Integration of the *binK* allele into the *att*Tn*7* site was verified using the primers Tn7 Site F and Tn7 Site R.

### Construction of pBinK.

A gene fragment containing the wild-type *binK* open reading frame, along with 300 bp upstream and 300 bp downstream, was PCR amplified using Pfx50 as described above with primers MJM-713 and MJM-714 and MJM1100 genomic DNA. The gene fragment was blunt cloned into the HpaI-digested vector pVSV104. The plasmid construct was sequenced using the primers JFB_355 through JFB_378.

### Construction of pM1422.

The *sypA*′-*gfp*^*+*^ transcriptional reporter was cloned by amplifying the ES114 *sypA* promoter with Pfx50 as described above using primers MJM-475F and MJM-476F and MJM1100 genomic DNA. The resulting PCR product was cut with XmaI and XbaI, and the XmaI-XbaI-cut product was introduced into the XmaI and XbaI sites of pTM267, respectively (32). The resulting plasmid, termed pM1422, was shown to have green fluorescent protein (GFP) activity regulated by RscS and to contain a wild-type ES114 sequence across the length of the insert.

### Construction of rscS* Δ*binK* strain.

A strain containing an in-frame deletion in *binK* and harboring the constitutive *rscS* allele was constructed using TfoX-based transformation as previously described (6, 33). The plasmid pLostfoX-Kan was introduced into the Δ*binK* (recipient) strain by conjugation. The recipient was grown overnight in LBS containing kanamycin and subcultured 1:100 into 3 ml of Tris minimal *N*-acetylglucosamine containing kanamycin and grown overnight with aeration. The recipient was subcultured again at 1:50 into 20 ml of Tris minimal *N*-acetylglucosamine containing kanamycin and grown with aeration to an optical density at 600 nm (OD_600_) of 0.25 to 0.30. DNA was isolated from the strain MJM1198, which harbors the marked rscS* allele, with the Qiagen DNeasy kit; 2.4 μg of donor genomic DNA was incubated with 500 μl of prepared recipient. Following a brief vortex, the samples were incubated statically at room temperature for 30 min. LBS (1 ml) was added, and the samples were allowed to recover overnight. Cells (50 μl) were plated onto LBS containing chloramphenicol to select for transformants. After transformation, loss of the pLostfoX-Kan plasmid was confirmed by ensuring that the resulting strain was sensitive to kanamycin.

### Construction of rscS* Δ*binK sypG*::Tn*erm* strain.

The marked *sypG* allele was transformed into MJM2255/pLostfoX-Kan (MJM2488) as described above with the following modifications: donor genomic DNA was prepared from strain MJM1954, which harbors the marked *sypG*::Tn*erm* allele, and transformants were selected on LBS containing erythromycin (6, 33).

### Competition assays *in vitro*.

The Δ*binK* mutant and the wild-type strain MJM1100 (ES114) carrying plasmid pVSV103 that constitutively expresses LacZ (β-galactosidase) were grown overnight with shaking at 25°C in LBS and LBS containing kanamycin, respectively. Both of the overnight cultures were diluted 1:80 in LBS and grown at 25°C with shaking to an OD_600_ of 0.2 to 0.3. The two strains were normalized by optical density and mixed at a 1:1 ratio (34). The mixed inoculum was diluted 181-fold in LBS in triplicate, allowed to grow at 25°C with shaking for 7.5 generations, diluted 181-fold again and grown for another 7.5 generations, for a total of 15 generations, and plated. The blue/white ratios were used to score these competitions as done previously.

### Competition assays *in vivo*.

The Δ*binK* mutant and the wild-type strain MJM1100 (ES114) carrying plasmid pVSV103 that constitutively expresses LacZ (β-galactosidase) were grown overnight with shaking at 25°C in LBS and LBS containing kanamycin, respectively. Both of the overnight cultures were diluted 1:80 in LBS and grown at 25°C with shaking to an OD_600_ of 0.2 to 0.3. The two strains were normalized by optical density and mixed at a 1:1 ratio (34). *E. scolopes* hatchlings were exposed to mixed inoculum concentrations of 2 × 10^3^ CFU/ml for 3 h. Then, squid were transferred to 40 ml of uninoculated filter-sterilized Instant Ocean (FSIO) for an additional 45 h (water was changed at 24 h postinoculation), at which point they were euthanized and surface sterilized by storage at −80°C. Each squid was homogenized and plated, and the blue/white colony ratios were used to score these competitions as done previously (6, 35). In these experiments, all squid were colonized by both strains in the competition.

### Squid colonizations for CFU counts.

*E. scolopes* hatchlings were colonized by exposure to approximately 2 × 10^3^ CFU/ml of each strain in a total volume of 40 ml of FSIO for 3 h. Squid were then transferred to 40 ml of uninoculated FSIO for an additional 45 h (water was changed at 24 h postinoculation), at which point they were euthanized and surface sterilized by storage at −80°C according to standard procedures (35). For determination of CFU per light organ, each squid was thawed and homogenized, and 50 μl of each homogenate was plated onto LBS plates. Bacterial colonies from each plate were counted and recorded.

### Aggregate assessment.

*E. scolopes* hatchlings were exposed to inoculum concentrations of 2 × 10^6^ CFU/ml for 3 to 5 h prior to dissection. The juvenile squid were anesthetized in FSIO containing 2% ethanol. Each squid was placed ventral side up on a depression well slide and dissected to expose the light organ. GFP-labeled (green) bacteria were viewed using a Nikon Eclipse 90i fluorescence microscope with appropriate filter sets. Aggregate sizes were assessed using the NIS-Elements Advanced Research version 3.2 autodetection tool, which calculates area measurements in square micrometers based on views with increased fluorescent intensity.

### Wrinkled colony assay.

Cultures were grown overnight in LBS with shaking at 25°C. Eight-microliter aliquots were spotted onto LBS, LBS containing tetracycline, or LBS containing tetracycline and kanamycin, as required for plasmid maintenance. Plates were incubated at 25°C, 28°C, and 30°C for 48 h. Colonies were imaged on the Leica Firecam microscope at 48 h postspotting. For the time course assay, plates were incubated at 25°C, and colonies were imaged at the indicated time points postspotting.

### Syp exopolysaccharide immunoblotting analysis.

Exopolysaccharide (EPS) from V. fischeri cells was isolated. Antiserum raised against Syp EPS was first blocked with non-EPS-producing V. fischeri ES114 and then used for immunoblotting against the isolated EPS, as described in detail previously (36, 37).

### *syp* expression analysis.

Bacterial strains harboring the pM1422 plasmid were grown overnight with shaking at 25°C, 28°C, and 30°C. A 400-μl aliquot of each overnight culture was spun down in a centrifuge at 6,000 × *g* for 10 min. The supernatant was decanted, and each pellet was resuspended in 100 μl of FSIO. Each resuspension was arrayed in a 96-well flat-bottom Costar plate. The fluorescence of each well was read using 485 nm excitation/535 nm emission for the GFP and 535 nm excitation/612 nm emission for the mCherry sequentially on the Synergy H1 hybrid multi-mode microplate reader (BioTek).

### Growth curves.

Bacterial microplate growth curves were obtained as described previously (6). Strains were grown overnight with shaking at 25°C and arrayed in a 96-well plate. The master plate, containing glycerol as a cryoprotectant, was frozen at −80°C. For growth curves, the master plate was thawed on ice, and 1 μl was pin replicated into a new 96-well plate containing 100 μl of fresh LBS per well; cultures in the new plate were grown overnight at 25°C with aeration. A new 96-well plate was prepared with 99 μl of LBS per well, into which 1-μl amounts of the overnight cultures were pin replicated. The inoculated plate was incubated in a Synergy H1 hybrid multimode microplate reader at 25°C, 28°C, or 30°C. The OD_600_ of each well was recorded every 10 min for 30 h with a 5-min period of shaking every 10 min. To prevent condensation on the lids, each lid was precoated for 30 min with 10 ml of 0.05% Triton X-100 in 20% ethanol and then air dried.

## RESULTS

### The hybrid histidine kinase BinK is a negative regulator of squid colonization.

**FIG 1:**
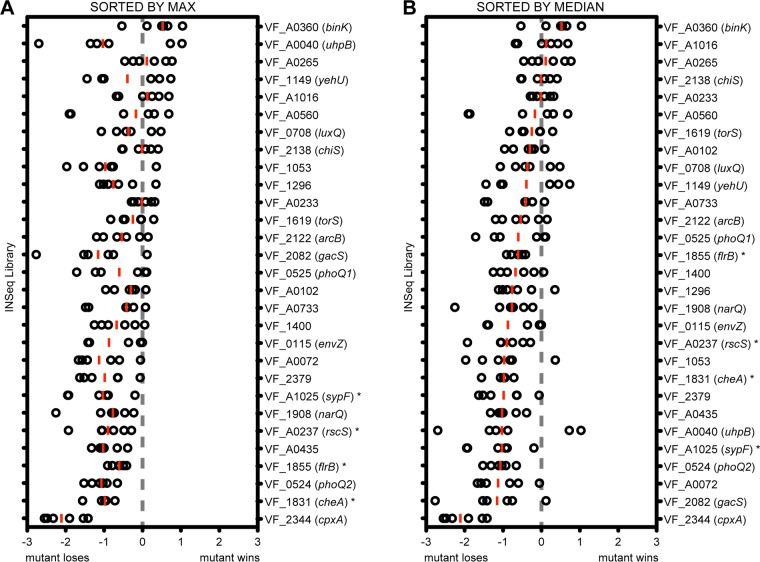
Histidine kinase mutant dynamics during colonization in an INSeq experiment. Colonization data for the 29 histidine kinases that were sampled *in vivo* in our previous INSeq study (6). Circles represent INSeq analysis of 250 squid each, and the log ratio is computed as in the previous study. Red bars represent the median replicate for each mutant. Genes indicated by an asterisk are ones for which there was prior evidence for a role in squid colonization. The same data are sorted by their maximum (A) or median (B) as described in the text.

Two-component signaling systems are important mediators of bacterial interaction with their environment, including animal host niches. A previous study in V. fischeri identified response regulators that affect squid colonization, but there has not been a comparable examination of V. fischeri histidine kinases (HKs) (17). We recently completed a global investigation of bacterial mutant behavior during squid colonization using insertion sequencing (INSeq) technology, and here we have proceeded to examine the distribution of histidine kinases across the colonization data set (6). Gene products for which the corresponding mutants exhibited a substantial colonization defect included sensors in known colonization pathways. These included the Syp biofilm kinases RscS and SypF and the flagellar motility and chemotaxis kinases FlrB and CheA (Fig. 1). There was one HK, CpxA, for which the mutant yielded a more severe colonization defect, but follow-up analysis revealed it to have a growth defect *in vitro*, and, therefore, we did not pursue it in this analysis (6).

**FIG 2:**
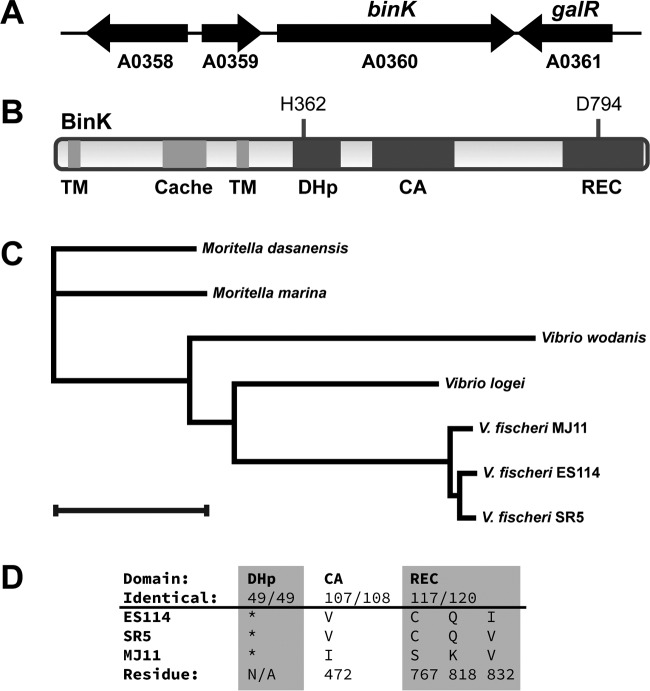
*binK* locus and BinK domain structure. (A) The *binK* locus. Locus tag “VF_” suffixes (e.g., VF_A0360) are shown, along with gene names where assigned. VF_A0358 encodes a predicted acriflavin resistance periplasmic protein, VF_A0359 encodes a predicted TetR family transcriptional regulator, and VF_A0361 is *galR*, which encodes a predicted galactose-responsive DNA-binding transcriptional repressor. (B) Predicted domain structure of BinK, including the transmembrane (TM) domains, the periplasmic loop that includes a Cache domain, and the cytoplasmic two-component protein signaling domains: dimerization and histidine phosphotransferase (DHp), catalytic and ATPase (CA), and receiver (REC). (C) Neighbor-joining tree of BinK orthologs. Bar, 0.1 distance metric. Proteins are from V. fischeri ES114 (GenBank accession AAW87430.1), V. fischeri SR5 (EHN69059.1), V. fischeri MJ11 (ACH63581.1), Vibrio *logei* (RefSeq accession WP_035470791.1), Vibrio wodanis (WP_045104527.1), Moritella dasanensis (WP_026006314.1), and Moritella marina (WP_019439447.1). (D) DHp domains among the V. fischeri orthologs are identical (*), and CA and REC domains are identical except for the residues listed.

**FIG 3:**
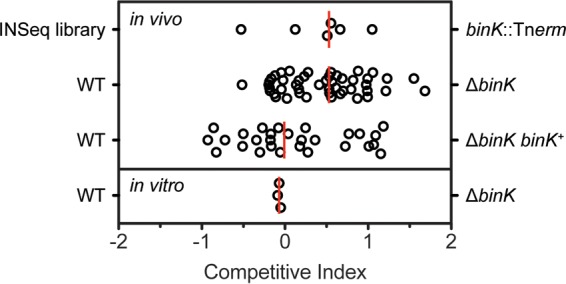
Mutants lacking BinK exhibit an advantage when competing directly against the wild-type parent strain. The top row (INSeq library) shows the same data as Fig. 1 for reference. The other rows represent competition experiments in which two strains were coinoculated and permitted to colonize squid (*in vivo*) or were coinoculated into culture medium and grown for a corresponding number of generations (*n* = 15). Individual samples (squid or culture tubes) are plotted as circles, and medians from at least three biological replicates are plotted as red lines. The competitive index is equal to the log-transformed value of the mutant/wild-type ratio after competition normalized to its measured ratio at the beginning of the competition. All of the animals were colonized by both strains. Competition of wild-type ES114 with the isogenic Δ*binK* strain shows the 4-fold advantage of the mutant. This advantage is not observed upon complementation with *binK*^+^ at the *att*Tn*7* site or *in vitro*.

At the other end of the distribution, there appeared to be genes for which disruption was predicted to enhance competitive squid colonization. In contrast to the large number of studies on mutants with colonization defects in V. fischeri, there are only a small number that describe mutants that exhibit enhanced colonization (38, 39). As a result, we did not have a strong data set on which to train our methods for identification of new negative regulators of colonization. To determine whether the INSeq screen identified mutants with bona fide competitive advantages in the squid host environment, we selected the gene with the largest such phenotype for examination and characterization in this report, an orphan hybrid histidine kinase encoded by *binK* (Fig. 1). Mutations in *binK* were not predicted to be polar on a downstream gene, and BinK encoded domains typical of a hybrid histidine kinase that signals through an HPt domain-containing protein and a response regulator (Fig. 2). In fact, BinK is encoded in all three sequenced V. fischeri genomes; the predicted phosphorylation sites are conserved, and the annotated domains have at least 97% identity (Fig. 2C and D). The *binK* mutant was predicted to have a dramatic colonization advantage in the INSeq study: among INSeq library-colonized replicates of 250 squid, *binK* mutants exhibited a median 4-fold enrichment compared to their abundance in the library prior to colonization. To test whether this phenotype would be observed in a more controlled assay, we constructed a Δ*binK* deletion strain and performed competitive colonization of the mutant against that of the wild-type strain ES114. In this assay, the mutant exhibited a similar phenotype as predicted from the global analysis, and the median competitive advantage for the mutant was 4-fold (Fig. 3). The Δ*binK* strain did not exhibit a competitive advantage in culture, providing evidence that the observed advantage *in vivo* was specific to the host environment (Fig. 3). Introduction of a wild-type allele of *binK* at the Tn*7* site complemented the mutant, supporting the fact that the observed phenotypes are attributable to BinK and that the presence of BinK reduces competitive colonization fitness. Therefore, BinK is a putative negative regulator of squid colonization. A major goal of this study was to identify a negative regulator from the INSeq data set and examine how it performed in controlled competition assays. The competition data obtained here confirmed that the *binK* mutant reproducibly outcompeted the isogenic wild-type strain. We therefore proceeded to identify the developmental stage at which BinK influences colonization.

### BinK is a negative regulator of *in vivo* aggregation.

**FIG 4:**
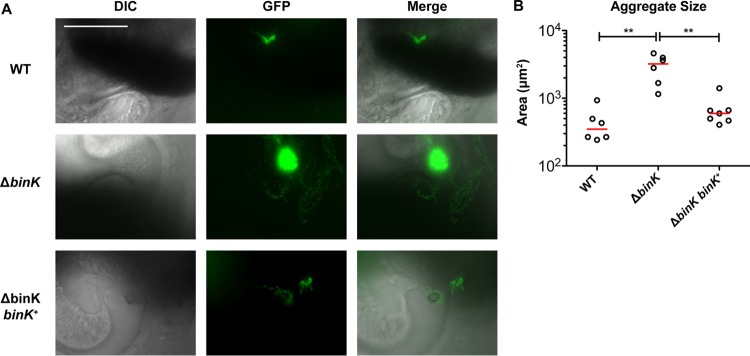
BinK negatively regulates aggregation in the squid mucus. Imaging of bacterial aggregates in host mucus by differential interference contrast (DIC) and epifluorescence microscopy. V. fischeri cells (4 × 10^5^ to 6 × 10^5^ CFU/ml) constitutively expressing GFP from the pVSV102 plasmid were inoculated into filter-sterilized Instant Ocean containing juvenile squid and imaged at 3 to 5 h postinoculation. (A) Bar, 100 μm; all panels are at the same scale. (B) Quantification of the area of the aggregates shows a significant BinK-dependent effect. WT, wild type.

The earliest stages of bacterial colonization can be directly imaged in *E. scolopes* squid juveniles. Newly hatched squid acquire bacteria from the seawater, and the colonizing V. fischeri aggregate on cilia within host-produced mucus (8, 40). Furthermore, aggregation is known to be critical for proper colonization (9, 10). Using constitutive green fluorescent protein (GFP) expression to mark colonizing bacteria, we examined aggregation behavior in wild-type V. fischeri and in strains lacking BinK. GFP-expressing wild-type cells exhibited consistent aggregation 3 to 5 h postinoculation (hpi) against the ciliated appendages of the host light organ (Fig. 4). Aggregation of Δ*binK* occurred in a comparable time frame but with many more bacterial cells, resulting in a larger biofilm-like aggregate. The complemented *att*Tn*7*::*binK*^*+*^ strain exhibited aggregation comparable to that of the wild-type strain. Quantification of the area bounded by bacterial fluorescence in aggregates in at least *n* = 6 replicates per sample was plotted (Fig. 4B), which demonstrated that across animals BinK negatively regulates aggregate size in the squid host during the critical stage of colonization initiation.

### BinK is a negative regulator of Syp biofilm phenotypes in culture.

**FIG 5:**
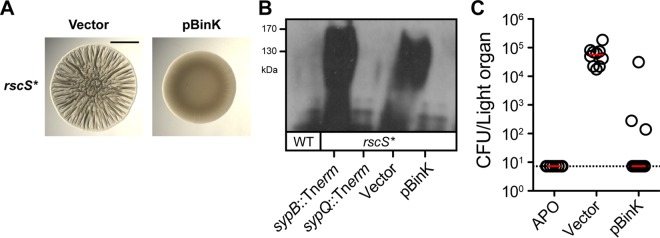
Overexpression of BinK inhibits biofilm formation and squid colonization. (A) Wrinkled colony formation in the rscS* strain MJM1198 is inhibited upon overexpression of BinK. Bar, 0.5 cm. (B) Immunoblot analysis of Syp exopolysaccharide (EPS) in strain ES114 and in MJM1198-derived rscS* strains. Vector is pVSV104, the parent for the pBinK plasmid. The *sypB* and *sypQ* mutants function as controls. SypQ is required for EPS production, whereas SypB is specifically required for EPS secretion. (C) The effect of BinK overexpression on squid colonization. V. fischeri strain ES114 cells (2 × 10^3^ CFU/ml) carrying the pVSV104 vector control or the pBinK derivative were inoculated into filter-sterilized Instant Ocean containing juvenile squid and assessed for CFU at 24 h postinoculation. The limit of detection is shown by the dotted line, and the median of each data set is shown by the red bar; the data were collected alongside those for control uncolonized animals (APO, aposymbiotic).

V. fischeri aggregation in the host is dependent on the hybrid histidine kinase RscS and its 18-gene target locus *sypABCDEFGHIJKLMNOPQR* (25, 26, 41). There is a strong correlation between phenotypes observed in the host and those in the colony biofilm assay, in which a biofilm-induced strain forms polysaccharide-rich rugose colonies on rich medium agar plates (37, 42). Unless noted otherwise, for colony biofilm formation, we typically employ the *rscS*-overexpressing strain MJM1198, which we denote rscS* (6, 37). We proceeded to ask whether overexpression of BinK alone was sufficient to interfere with biofilm formation in such an assay. Expression of BinK from a medium-copy vector (pVSV104 backbone [34]) led to complete inhibition of wrinkled colony phenotypes when assayed 48 h after plating (Fig. 5A). When polysaccharide production was assessed with an antibody against the Syp polysaccharide, overexpression of BinK was sufficient to reduce the levels of Syp polysaccharide below detection, even in this otherwise biofilm-induced background (Fig. 5B). For squid colonization of strains overexpressing BinK, we observed a substantial deficit (more than 3 orders of magnitude) in the number of bacteria recovered from the host, and in most instances, colonization was below our limit of detection (Fig. 5C). These data argue for a role of BinK in regulating the production of Syp polysaccharide and support assignment of BinK as a negative regulator of biofilm formation and squid colonization.

**FIG 6:**
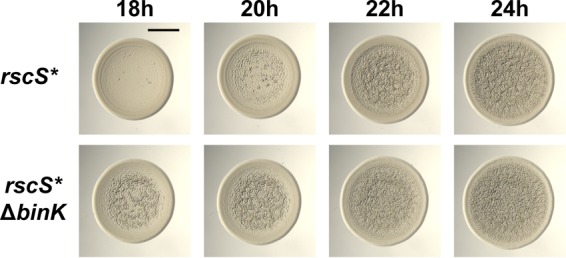
The Δ*binK* strain exhibits accelerated wrinkled colony formation. Time course assay of wrinkled colony formation in the rscS* strain (MJM1198). Overnight cultures of the indicated strains were spotted onto LBS medium at room temperature, and wrinkled colony formation was imaged 18 to 24 h postspotting. Bar, 0.5 cm.

The standard wrinkled colony assay relies on a biofilm-induced genetic background that permits visual inspection of the phenotype on the agar surface. Characterization of positive regulators of wrinkled colony formation is straightforward in that mutants in such factors have wrinkling that is delayed or completely absent (25, 37). The colony biofilm phenotype is especially strong, and, therefore, nuanced approaches are required to examine negative regulation. For example, a kinetic approach has been used to reveal positive and negative activities within the response regulator SypE (43). We undertook a similar analysis. The rscS* strain MJM1198 begins to show evidence of wrinkling 19 to 20 h after preparation of 8-μl spots, whereas deletion of *binK* leads to an acceleration in the wrinkling phenotype (Fig. 6). Robust wrinkling in the mutant is already present by 18 h, consistent with BinK playing a role in inhibiting biofilm formation (Fig. 6).

**FIG 7:**
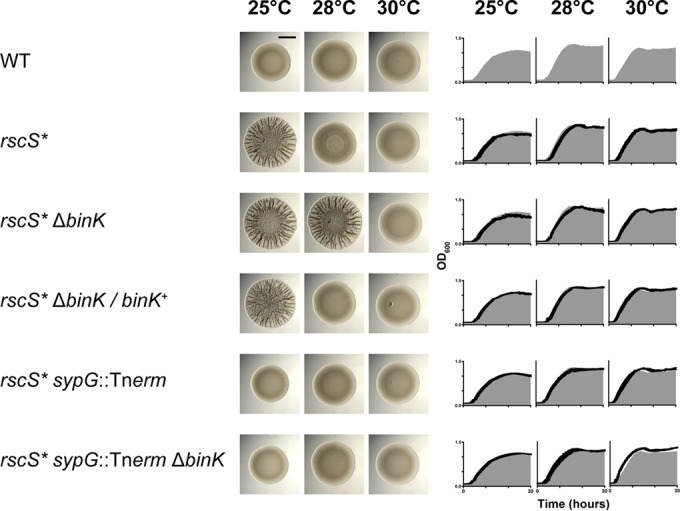
BinK is a negative regulator of Syp biofilm phenotypes. Wrinkled colony formation was assessed at 25°C, 28°C, and 30°C in the strains indicated. Only the Δ*binK* mutant produced a wrinkled colony at both 25°C and 28°C. Wrinkled colony formation in the Δ*binK* mutant was dependent on SypG. Growth curves were obtained for the strains indicated at each of the three temperatures assessed, with the wild-type (WT) curve for each temperature represented by the gray area. Images were taken 48 h postspotting.

There were hints in the literature that overexpression of *rscS* induces a robust colony biofilm at 25°C but not at 28°C (i.e., from an rscS* allele carried on the plasmid-borne pKG11). To assess the temperature phenotype directly and to determine whether BinK contributed to a temperature-dependent effect, we assessed wrinkled colony formation and observed a prominent biofilm at 28°C only in the absence of BinK (Fig. 7). As expected, wrinkled colony formation was dependent on SypG (Fig. 7). We did not observe any significant effect of BinK on bacterial growth. These results argue that in the absence of BinK, biofilm signaling from RscS is derepressed at 28°C.

### BinK influences syp gene transcription.

**FIG 8:**
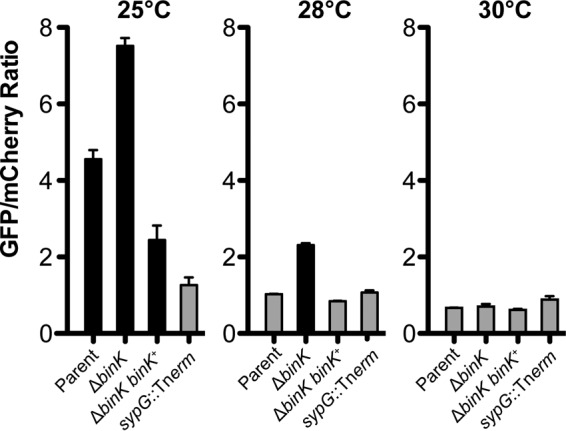
BinK is a negative regulator of *sypA*′-*gfp*^*+*^ at 25°C and 28°C. A *sypA*′-*gfp*^*+*^ transcriptional reporter was assessed in cultures grown at 25°C, 28°C, and 30°C. Strains that produce a wrinkled colony on LBS are shaded in black. The parent strain is MJM1198. The experiment was performed in triplicate, and error bars represent the standard errors of the mean.

SypG is a σ^54^-dependent activator of syp locus transcription (26, 44). There are characterized regulators that act upstream of SypG to influence syp transcription (e.g., RscS and SypF) and factors that act downstream of SypG to influence exopolysaccharide production by other means (e.g., SypE/SypA and DnaJ) (6, 24, 45). We therefore asked whether syp transcription was affected by the absence of BinK. We constructed a *sypA*′-*gfp*^*+*^ transcriptional fusion and assessed GFP activity in a strain lacking BinK. The reporter was constructed in the pTM267 backbone, in which constitutive mCherry (red) fluorescence normalizes for plasmid copy number, and the resulting transcriptional activity from the test promoter (green fluorescence) can be determined with high precision (32). Using this reporter, we noted that in the parent strain higher GFP levels from the *sypA*′-*gfp*^*+*^ fusion were observed at 25°C than at 28°C, consistent with the wrinkled colony phenotypes at the two temperatures. Strains lacking BinK exhibited elevated *sypA*′-*gfp*^*+*^ activity, at both 25°C and 28°C (Fig. 8). During the course of these assays, we additionally examined a higher temperature to determine what effect additional perturbations would have on syp expression. At 30°C, we observed that all samples had baseline levels of *sypA*′-*gfp*^*+*^ activity, including those lacking BinK. We examined the morphology of these strains and found that the spotted colonies were completely smooth (Fig. 7). Therefore, *sypA*′-*gfp*^*+*^ expression correlates with wrinkled colony biofilm formation across a broad set of temperature and mutant conditions, supporting a role for BinK in regulating Syp biofilm through its regulation of syp transcription.

### BinK signals even in the presence of a locked SypE-SypA pathway.

**FIG 9:**
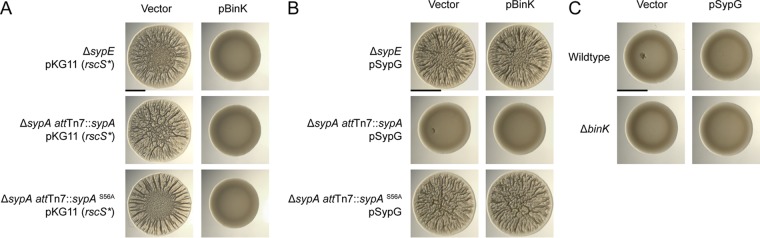
pSypG is epistastic to BinK, and BinK signaling is independent of SypE-SypA. (A) Wrinkled colony formation in the Δ*sypE*, Δ*sypA att*Tn*7*::*sypA*, and Δ*sypA att*Tn*7*::*sypA*^S56A^ strains carrying the plasmid pKG11 (rscS*) is inhibited upon overexpression of BinK. Wrinkled colony formation was assessed on LBS-tetracycline (Tet)-kanamycin (Kan). (B) Wrinkled colony formation in the Δ*sypE*, Δ*sypA att*Tn*7*::*sypA*, and Δ*sypA att*Tn*7*::*sypA*^S56A^ strains carrying the plasmid pSypG (pEAH73) is not inhibited upon overexpression of BinK. Wrinkled colony formation was assessed on LBS-Tet-Kan. (C) Wrinkled colony formation is not observed in the wild-type or Δ*binK* strains carrying the plasmid pSypG (pEAH73). Wrinkled colony formation was assessed on LBS-Tet. All images were taken 48 h postspotting. Bars, 0.5 cm.

The above results suggested that BinK signals at or upstream of SypG and that BinK has an effect on SypG-dependent transcription of the syp locus. We note that RscS-SypF signaling leads to activation of SypG and inhibition of SypE (and subsequent activation of SypA). Both activation of SypG and activation of SypA are required to observe wrinkled colony formation (45). We therefore asked whether BinK acts to inhibit SypG and/or to signal through the SypE-SypA pathway. First, we found that in strains lacking SypE, overexpression of BinK (i.e., pBinK) still interferes with wrinkled colony formation (Fig. 9A). Similarly, locking the pathway in an activated state with a constitutive SypA allele did not interfere with the ability of pBinK to inhibit wrinkled colony formation (Fig. 9A). We note that the rscS* allele in this panel is different, a plasmid-borne pKG11 (25), but this has no effect on the BinK phenotypes and provides additional assurance that the effects are general and not due to one specific induction system.

We next changed to a system in which biofilm induction is accomplished directly by overexpression of SypG (i.e., pEAH73 [46]; here called pSypG for clarity). In the absence of SypE, pSypG stimulates wrinkled colony formation (Fig. 9B). In this background, BinK had no effect, suggesting that SypG overexpression is epistatic to BinK. This effect was independent of signaling through the SypE-SypA pathway (Fig. 9B). Furthermore, inactivation of wild-type SypA (by SypE; no wrinkling) and the constitutively active SypA^S56A^ (wrinkled colony) yielded the predicted phenotypes regardless of the presence of pBinK (Fig. 9B).

The above data support BinK signaling through SypG (but not SypE-SypA), though they are based on the BinK overexpression construct. We next asked whether deletion of *binK*, which leads to wrinkled colony formation in the rscS* strain MJM1198, similarly leads to wrinkled colony formation in strains carrying pSypG and an intact SypE (i.e., pSypG *sypE*^*+*^). If a Δ*binK* mutant exhibits wrinkled colony formation in a pSypG *sypE*^+^ background, then this result suggests that BinK can signal to dephosphorylate SypA. Instead, we observed that Δ*binK* had no effect in the pSypG *sypE*^+^ background (Fig. 9C). Therefore, the results in Fig. 9 consistently argue that BinK is acting through SypG and not SypE.

## DISCUSSION

In this report, we investigated the histidine kinase for which mutation revealed the greatest colonization advantage in an insertion sequencing study. This work further validated the global approach in the squid model, led to the identification of BinK as a novel colonization inhibitor, and revealed that BinK negatively regulates bacterial aggregation, Syp EPS, and syp gene transcription. We address these issues below.

### INSeq as a discovery tool for mutants that exhibit a competitive advantage.

In this report, we present BinK as the first validated negative regulator of host colonization from the INSeq global approach. In our previous study, use of INSeq as a discovery tool was limited to a detailed analysis of mutants that were deficient in colonization. To identify novel colonization-deficient mutants, we used 37 previously identified mutants that exhibited competitive defects to train the data set. In contrast, previously identified mutants that exhibited a colonization advantage were poorly represented in the global data set (*litR*) or previously exhibited various competition phenotypes during the first 48 h (*hnoX*) (38, 39). Examination of the *binK* data in the INSeq data set is likely to serve as a useful marker for identification of other relevant negative regulators during host colonization. The magnitude of the effect (approximately 4-fold colonization advantage for the mutant at 48 h) is among the largest advantage that has been observed in the system and is comparable to the median reported previously for a mutant of the quorum-sensing regulator LitR (38). The tight agreement between the median INSeq result and the median competition result observed in 1:1 competitions with the wild type (Fig. 3) suggest that for mutants overrepresented in the output pool median may be a strong predictor of their colonization in defined colonization assays. We have therefore reordered the histidine kinase mutants in the INSeq data set by median value in Fig. 1B. As additional such candidates are characterized, we will assess the predictive value of the INSeq results.

INSeq mutants defective for colonization were enriched for the “initiation” stage of colonization. Colonization initiation includes biofilm formation in the host, and more than 30% of the validated mutants affected biofilm phenotypes *in vitro* (6). In this study, we started with the INSeq data set and then examined a previously uncharacterized gene for which the mutant exhibited a colonization advantage. We think it significant that biofilm formation and colonization initiation are also represented among the first colonization negative regulators identified from the screen, and this further bolsters the importance of biofilm regulation for V. fischeri.

### BinK is highly conserved across V. fischeri strains.

V. fischeri forms tight aggregates in the squid mucus, and it seems likely that such behavior might be selected against during dispersal from the squid and in the ocean. Therefore, BinK inhibition of aggregation may provide a means for cells to remain planktonic when they are not undergoing acquisition by the squid host, both to facilitate access to nutrients and to enable uptake by another squid host. We note that BinK is conserved in the Mediterranean squid symbiont V. fischeri SR5, which colonizes squid yet lacks RscS (47, 48). Discovery of BinK presents the intriguing possibility that squid-specific symbionts lacking *rscS* promote syp expression by interfering with BinK activity. BinK is largely conserved in the fish symbiont MJ11, having 97% amino acid identity to ES114 (98% similarity) across the protein, with residues that differ in annotated signaling domains noted in Fig. 2D. It remains to be examined whether these sites have functional consequences for host colonization specificity.

### BinK is an inhibitor of syp-dependent biofilm formation.

We have shown that BinK negatively regulates Syp EPS production and that it represses syp transcription. It seems likely that kinase and/or phosphatase activity is required for BinK function, and future work will test the role of BinK during biofilm formation by both genetic and biochemical methods. The data presented here support the hypothesis that BinK antagonizes the signal from RscS and that BinK exerts this effect through the SypG arm of the pathway, independent of SypE and SypA. It might perform this function by directly regulating the phosphorylation status of SypG. As a predicted membrane-bound hybrid histidine kinase, BinK requires an HPt domain-containing protein and a response regulator receiver domain (REC) for signaling. SypF (HPt) and SypG (REC) are possible candidates, respectively, especially given that previous analyses of response regulators did not identify candidates involved in wrinkled colony formation outside those encoded in the syp locus (17, 46). We noted that deletion of *binK* did not phenocopy the Δ*sypE* allele. Therefore, either BinK signals once the SypG pathway has diverged from the shared (i.e., SypF) portion of the pathway or BinK signaling in the shared portion of the pathway (e.g., RscS or SypF) disproportionately influences SypG activity. RscS serves as a phosphate donor for SypF-SypG, so future work will test whether BinK acts to dephosphorylate SypF under planktonic conditions. In this model, inhibition of BinK phosphatase activity might lead to a redirection of phosphates to SypG, leading to syp gene activation. We have not ruled out the possibility that BinK antagonizes RscS by other means (alternate pathways or not via phosphotransfer), and testing of the ideas described here will provide a clearer picture of how BinK activity is modulated during colonization, during dispersal, and in the environment.

### Temperature influences biofilm development in V. fischeri.

We note that biofilm phenotypes in RscS-overexpressing strain MJM1198 are robust at 25°C, diminished at 28°C, and absent at 30°C. In many organisms, including Listeria monocytogenes, Yersinia pestis, Vibrio cholerae, and Pseudomonas aeruginosa, temperature has been noted to influence biofilm regulation (49–51). In many instances, there is a correlation between traits expressed at higher temperatures (e.g., 37°C) and pathogenicity in mammalian hosts; however, we point out that the squid host is ectothermic and does not internally regulate its body temperature. Therefore, a role for temperature regulation to discriminate the presence of this host is unlikely, recalling phenotypes observed in other invertebrate-associated microbes, e.g., bacterial colonizers and pathogens that influence coral bleaching pathogenesis (52, 53). In V. fischeri, at least one other temperature-responsive system, the heat shock chaperone DnaK-DnaJ has been shown to be required for bacterial aggregation *in vivo* and syp-dependent biofilm phenotypes *in vitro* (6). Taken together, these data suggest that the Syp biofilm can be experimentally manipulated through temperature regulation to reveal relevant regulation in the host. It will be interesting to examine whether the temperature phenotypes observed are representative of marine bacteria that live in an environment in which the temperature is variable or whether selection to resist host insults (i.e., reactive oxygen and nitrogen species) has manifested as a temperature-dependent biofilm phenotype.

Our previous work and studies from other groups have established biofilm formation as a critical developmental event during squid colonization. V. fischeri rapidly transitions from a single-celled planktonic lifestyle in seawater to an aggregated state in the host mucus. The molecular communication between the host and symbiont to control this lifestyle transition is poorly understood. This work has revealed a novel two-component histidine kinase that is important for biofilm signaling *in vivo*, bringing us closer to an understanding of the chemical communication at the microbe-host interface.
